# Altered motivation states for physical activity and ‘appetite’ for movement as compensatory mechanisms limiting the efficacy of exercise training for weight loss

**DOI:** 10.3389/fpsyg.2023.1098394

**Published:** 2023-04-28

**Authors:** Kyle D. Flack, Matthew A. Stults-Kolehmainen, Seth A. Creasy, Saumya Khullar, Daniel Boullosa, Victoria A. Catenacci, Neil King

**Affiliations:** ^1^Department of Dietetics and Human Nutrition, University of Kentucky, Lexington, KY, United States; ^2^Division of Digestive Health, Yale New Haven Hospital, New Haven, CT, United States; ^3^Department of Biobehavioral Sciences, Teachers College, Columbia University, New York, NY, United States; ^4^Division of Endocrinology, Metabolism, and Diabetes, University of Colorado Anschutz Medical Campus, Aurora, CO, United States; ^5^Anschutz Health and Wellness Center, University of Colorado Anschutz Medical Campus, Aurora, CO, United States; ^6^School of Applied Sciences, Edinburgh Napier University, Edinburgh, United Kingdom; ^7^Faculty of Physical Activity and Sports Sciences, Universidad de León, León, Spain; ^8^College of Healthcare Sciences, James Cook University, Townsville, QLD, Australia; ^9^Graduate Program in Movement Sciences, Integrated Institute of Health, Federal University of Mato Grosso do Sul, Campo Grande, Brazil; ^10^School of Exercise and Nutrition Sciences, Faculty of Health, Queensland University of Technology, Brisbane, QLD, Australia

**Keywords:** exercise compensation, exercise, physical activity, nonexercise activity thermogenesis, motivation, affectively charged motivation states, appetence, weight loss

## Abstract

Weight loss is a major motive for engaging in exercise, despite substantial evidence that exercise training results in compensatory responses that inhibit significant weight loss. According to the Laws of Thermodynamics and the CICO (Calories in, Calories out) model, increased exercise-induced energy expenditure (EE), in the absence of any compensatory increase in energy intake, should result in an energy deficit leading to reductions of body mass. However, the expected negative energy balance is met with both volitional and non-volitional (metabolic and behavioral) compensatory responses. A commonly reported compensatory response to exercise is increased food intake (i.e., Calories in) due to increased hunger, increased desire for certain foods, and/or changes in health beliefs. On the other side of the CICO model, exercise training can instigate compensatory reductions in EE that resist the maintenance of an energy deficit. This may be due to decreases in non-exercise activity thermogenesis (NEAT), increases in sedentary behavior, or alterations in sleep. Related to this EE compensation, the motivational states associated with the desire to be active tend to be overlooked when considering compensatory changes in non-exercise activity. For example, exercise-induced alterations in the wanting of physical activity could be a mechanism promoting compensatory reductions in EE. Thus, one’s desires, urges or cravings for movement–also known as “motivation states” or “appetence for activity”-are thought to be proximal instigators of movement. Motivation states for activity may be influenced by genetic, metabolic, and psychological drives for activity (and inactivity), and such states are susceptible to fatigue-or reward-induced responses, which may account for reductions in NEAT in response to exercise training. Further, although the current data are limited, recent investigations have demonstrated that motivation states for physical activity are dampened by exercise and increase after periods of sedentarism. Collectively, this evidence points to additional compensatory mechanisms, associated with motivational states, by which impositions in exercise-induced changes in energy balance may be met with resistance, thus resulting in attenuated weight loss.

## Introduction

Individuals classified as overweight or obese represent over 70% of US adults and over 50% of adults worldwide, putting an increasingly large portion of this population at risk for a multitude of comorbidities including cardiovascular disease, diabetes, heart disease, and certain cancers (Williams et al., 2015; Chooi et al., 2019). Obesity treatment has therefore emerged as a prime focus of health care and a top concern among the general public. Recent reports indicate 41.5% of individuals are currently trying to lose weight, with 65% of these individuals using exercise as at least one component of their weight loss strategy (Santos et al., 2017). Unfortunately, weight loss from exercise alone is often less than expected (Thomas et al., 2012, 2015). For instance, an individual may exercise to expend 2,000 kcal per week for 15 weeks, to total 30,000 kcal of exercise energy expenditure, although this rarely produces a body mass loss equivalent to 30,000 kcal during this time. Typically, individuals “compensate” for a portion of exercise energy expenditure in an effort to maintain energy homeostasis, thereby attenuating weight loss (King et al., 2007). Maintaining energy balance can be viewed as an evolutionarily conserved mechanism in place to retain bodily energy stores and preserve reproductive function, a useful survival strategy in times of reduced food availability (Rosenbaum and Leibel, 2010; Wang et al., 2019). Thus, humans may have a drive to compensate for energy expended in exercise even though this response is not advantageous in our current obesogenic environment. This has been highlighted in recent studies demonstrating similar total daily EE among individuals with differing levels of physical activity (Pontzer, 2018; Careau et al., 2021).

The degree to which individuals compensate in response to exercise is still unknown but may vary depending on the exercise stimulus. Some evidence indicates that exercise interventions featuring a greater energy expenditure (EE) evoke a greater compensatory response (Rosenkilde et al., 2012), supported by the observed lack of differences in weight loss between groups exercising at 50, 100 or 150% of public health recommendations (Church et al., 2007) or at 14 or 23 kcal/kg/week (Kraus et al., 2002). This is likely due to greater exercise EE eliciting a greater acute energy deficit and thus a stronger compensatory response to maintain energy homeostasis. In this light, the traditional “calories in, calories out” model, where increasing energy expenditure should result in greater losses in body mass, does not hold. On the other hand, others have demonstrated no differences in energy compensation between groups expending 3,000 or 1,500 kcal per week, or between groups exercising 6 days vs. 2 days per week, with all groups compensating roughly ~1,000 kcal per week (Flack et al., 2018, 2020a). One reason for these contrasting findings may be due to the high degree of inter-individual variability observed in the compensatory response to exercise, which is not fully understood (King et al., 2012).

Equivocal findings may also be explained by the variety of compensatory responses, independently and collectively, working to attenuate an exercise-induced energy deficit, reducing the likelihood of expected weight loss. This concept of energy compensation with exercise was initially recognized over 40 years ago by leading behavioral science and nutrition researchers Len Epstein and Rena Wing who noted, *“Exercise may stimulate the appetite so that persons who exercise increase their eating and do not lose as much weight as expected*” (Epstein and Wing, 1980). This hypothesis has been a focal point of attention among metabolism researchers over the past several decades, with many citing the Law of Thermodynamics of Homeostatic Control which states that an energy deficit induced by exercise will be balanced by an increased drive to eat to maintain energy balance. However, this assumes that energy intake (EI) is the only response available to restore homeostasis. King and colleagues defined specific compensatory responses, including automatic and volitional types versus metabolic and behavioral types, for both sides of the energy balance equation–more commonly for energy intake (EI) but also for energy expenditure (EE) (King et al., 2007). All these factors appear to have a place in the phenomenon of energy compensation in response to exercise, but none have adequately explained weight loss outcomes. It may be that the effect of exercise on weight loss is predicted by a combination of the type, magnitude, and temporal profile of these compensatory responses. These responses are also very specific to an individual, where not only may the magnitude of compensation be different, but the proportion of energy compensated through EE vs. EI may be different between individuals.

Compensation for exercise-induced energy deficits can be categorized, as noted above, into behavioral or metabolic responses, that can be either automatic or volitional (King et al., 2007). An automatic compensatory response can be considered a biological inevitability and obligatory, that is, not under conscious control by the individual. Volitional compensatory responses, on the other hand, are those under conscious control by the individual, requiring deliberate action. Most behavioral responses are volitional, such as changing our eating behaviors to increase EI, although some may be automatic and not under one’s conscious control, such as decreases in spontaneous physical activity (SPA), reflecting an unconscious drive for movement (Brand and Ekkekakis, 2018; Stults-Kolehmainen, 2022). Metabolic compensatory responses are processes designed to protect against continued weight loss by reducing metabolic EE (often expressed as kcal/kg body mass). All metabolic compensatory responses are automatic, as an individual cannot deliberately alter metabolic processes to change energy expenditure (such as resting metabolic rate). In this example of altering metabolic energy expenditure, an individual may attempt to prevent losses in lean mass during weight loss by consuming a diet high in protein, which could prevent declines in total resting energy expenditure; however, when adjusting energy expenditure for body weight or lean mass, declines in metabolic energy expenditure still persist (Rosenbaum et al., 2003; Goldsmith et al., 2010). Additional examples of metabolic and behavioral compensatory responses and their classification (automatic vs. volitional) are listed in Table 1. Such metabolic and behavioral compensatory processes that defend against a negative energy balance likely go back to our hunter-gatherer days, when food was scarce, and a large amount of activity-induced EE was an obligatory behavior to get food (Chakravarthy and Booth, 2004). Of highest relevance to this review are the volitional, behavioral compensatory responses (e.g., food intake and decreases in non-exercise physical activity) that tend to be deliberate and driven by motivational cues. Processes of automatic motivation, however, are also of interest. Of course, each of these behavioral responses could manifest in several facets of each behavior. For example, EI is reflected by the volume, energy density, and/or frequency of food consumption, while behavioral EE alterations could be conceptualized as changes in exercise volume or intensity, sleep duration, amount of time participating in sedentary activities, or metabolic alterations that reduce resting and non-resting energy expenditure. For both EI and EE associated compensatory responses, the temporal signaling is of importance. That is, acute bouts of exercise could immediately induce EI and EE compensatory responses, before any changes in tissue stores occur (Flack et al., 2022). In the event of a prolonged, marked negative energy balance, a different set of signals could promote behavioral compensatory responses due to loss of tissue stores (e.g., glycogen, lean mass, and adipose tissue).

**Table 1:** Metabolic and behavioral compensatory responses as varying by automaticity and volitional behavior*.

	Metabolic	Behavioral
**Automatic**	RMR	Spontaneous/Incidental activity	EE of exercise/PA	
**Volitional**	[None]	EI		Lifestyle PA

*Adapted from (King et al., 2007).

RMR, resting metabolic rate; EE, energy expenditure; EI, energy intake; PA, physical activity.

### Hypothesis – Motivation as a behavioral compensatory mechanism

It is generally accepted that increases in EI present to counter an exercise-induced energy deficit, although less is known regarding how changes in non-exercise, volitional EE present to counter this deficit or the mechanisms behind this response. In the search for underlying mechanisms that may drive energy compensation for exercise EE there has been a re-emerging interest in the study of motivation and how it relates to energy balance. Homeostasis of energy balance, including eating (Ferrario et al., 2016) and physical activity, are related to strong motivational factors (Stults-Kolehmainen et al., 2020; Budnick et al., 2022). These are generally controlled by two different systems, a homeostatic system and a hedonic system (Hussain and Bloom, 2013). Some factors are automatic and unconscious or barely noticeable (Ferrario et al., 2016); others, such as hunger, appetite and cravings, are perceptible and often even impossible to ignore (Hussain and Bloom, 2013). While all of these have been investigated for a considerable amount of time (Blundell and Cooling, 2000; Blundell et al., 2010), there is a new science of understanding them as a class of sensations – affectively-charged motivation states (ACMS) (Kavanagh et al., 2005). ACMS include urges, desires, and wants – basic intermediaries for motivated behaviors, such as eating, but also exercise and movement. Nevertheless, while these factors have been explored extensively on the EI side of the EB equation, they have not been commonly investigated on the EE side, and thus represent what could be an important gap in our understanding of motivation-driven processes influencing compensation. The present hypothesis paper will delve into aspects of the compensatory response to an exercise-induced energy deficit, specifically focusing on how the human body reduces non-exercise energy expenditure through mechanisms related to motivation to conserve energy and restore energy balance. We specifically hypothesize that compensation might be, in part, due to alterations in one’s appetence (or appetite) for movement and physical activity (Ferreira et al., 2006), or alternatively sedentarism and sleep, perhaps similar to changes that have been observed in the hedonic desire for highly-palatable food with exercise training.

## Discussion

### Understanding exercise, physical activity, sedentary behaviors, and the 24-h activity cycle

It is important to note the distinctions between exercise, physical activity (PA), and similar concepts. The classic taxonomy by Caspersen et al. (1985) states a difference between PA and exercise, such that PA is any movement eliciting an EE higher than basal levels, whereas exercise is a form of PA which is purportedly programmed and organized to be repeated over time to achieve specific goals. In other words, exercise interventions are designed to change different facets of physical fitness, which include physical and functional qualities, such as endurance, muscular strength, etc., but also body composition. PA not in the form of exercise is considered non-exercise activity thermogenesis, or NEAT (Levine, 2004). While these differentiations are theoretically clear and simple, few studies target both exercise and the superordinate category of PA in their analysis (Hautala et al., 2012; Tonello et al., 2015; Benítez-Flores et al., 2019). The lower number of studies may be due to conceptual pitfalls and methodological constraints. Nevertheless, some interesting results from these studies suggest that PA and exercise may interact with each other to potentiate or reduce physical fitness adaptations and health outcomes (Coyle et al., 2022). PA stands also in contrast to sedentary behavior or physical inactivity, which is not simply the opposite of PA, but rather they are asynchronous with each other. It is possible to be both very sedentary and very active on the same day (Thompson and Batterham, 2013; Raichlen et al., 2020). The 24-h activity cycle framework has been proposed as a new paradigm to integrate all forms of PA into one conceptual model (Rosenberger et al., 2019). However, it does not fit well with the classic taxonomy by Caspersen et al. (1985) as it only includes the four activities of sleep, sedentary behavior, light intensity physical activity (LIPA), and moderate-to-vigorous physical activity (MVPA), which have independent and interactive effects to simultaneously affect health. In other words, there may be positive and negative synergies among exercise, NEAT, sedentary time, and sleep. For instance, better sleep quality and/or quantity may result in greater PA the next day and greater PA may also result in better sleep (Master et al., 2019). Therefore, it is necessary to monitor the quality and quantity of any form of PA, including exercise, sedentary behaviors, and sleep, to better understand the relative influence of each activity component on health outcomes in bodyweight management interventions.

### Models of compensation – CICO, constrained total EE

Most studies on energy compensation with exercise have focused on EI, largely due to the accepted belief that EI is the largest source of compensation when exercising for weight loss (King et al., 2007; Thomas et al., 2012, 2015; Flack et al., 2018, 2020a). Although in the CICO model, the ‘calories out’, aspect cannot be overlooked, manifesting as decreases in EE in an attempt to negate an exercise-induced negative energy balance. As noted above, mechanisms working to decrease EE can be either physiological (decreases in resting energy expenditure (REE)/metabolic) or behavioral (decreases in PA outside the exercise intervention). These responses fit into the constrained total EE hypothesis speculating that in the presence of increasing levels of PA, total daily EE plateaus (Pontzer, 2015). There are several sources of cross-sectional data that support this hypothesis (Pontzer et al., 2012, 2016; Willis et al., 2022); however, prospective data are limited. In one study by Westerterp et al. (1992) previously sedentary males began training for a half marathon and had marked increases in total daily EE at 8 and 20 weeks; however, these increases in total daily EE tapered at week 40 even with an increase in exercise load. In females, total daily EE increased at week 8 but leveled off at weeks 20 and 40 even with increases in exercise load. However, the constrained model of compensation may not apply to individuals who purposely match additional daily EE with additional EI, such as in an athletic population not attempting to lose body weight (and often attempting to gain body weight). Maintaining energy balance via a high energy flux in this scenario would likely attenuate metabolic compensation (Bell et al., 2004).

### Changes in NEAT

What remains lacking in the constrained Total EE model is what specific components of EE are compensating in response to increases in PA. In controlled exercise studies, the EE of exercise is measured and held constant, leaving only resting and non-exercise EE to serve as compensatory variables. Some evidence suggests that REE, adjusted for body composition, may decline with increased aerobic exercise EE (Westerterp et al., 1992; Byrne and Wilmore, 2001); however, not all studies have demonstrated this effect (Donnelly et al., 2013; Creasy et al., 2022b). In addition to REE, a decline in non-exercise EE (or NEAT) is a likely mechanism of EE compensation. Reductions in NEAT can stem from improvements in skeletal muscle efficiency that reduce the energy cost of non-exercise activities (Rosenbaum et al., 2003; Goldsmith et al., 2010). Reductions in NEAT have also been attributed to decreases in non-exercise PA, including spontaneous (incidental) physical activity, such as fidgeting. The importance of the contribution of NEAT to energy balance and weight control was originally highlighted by Levine where NEAT has been proposed as a cause of differences in weight change in response to exercise interventions (Levine et al., 1999; Levine, 2004). It is important to note that Levine’s proposal of NEAT is based predominantly on cross-sectional data and hypothetical inferences. However, evidence for a decrease in NEAT comes from a carefully controlled, 7-day exercise intervention study by Stubbs et al. (2002). Participants tended to restore energy balance (EB) at a rate of 0.3–0.6 MJ/d by appearing to progressively reduce daily EE across 7 days. Several other studies suggest that initiating an aerobic exercise program leads to increases in sedentary behavior and decreases in non-exercise physical activity (NEPA) (Morio et al., 1998; Meijer et al., 1999; Di Blasio et al., 2012; Willis et al., 2014). Specifically Meijer et al. (1999) found that non-exercise activity measured with accelerometry was reduced on exercise training days compared to non-training days. In addition, a secondary analysis of the Midwest Exercise Trial 2 (MET-2), Herrmann et al. (2015) found that compensators (i.e., individuals with <5% weight loss) decreased non-exercise physical activity, whereas, non-compensators (i.e., individuals with ≥5% weight loss) increased non-exercise physical activity across a 10-month aerobic exercise intervention. This compensation may contribute to the declines in non-exercise EE noted above, and subsequently attenuate weight loss. Although results have been inconsistent, as indicated in a 2018 meta-analysis demonstrating 15 studies out of 36 reporting declines in NEAT (eight), NEPA (four) or both (three), pointing to the individual variability observed in this compensatory response. This meta-analysis also points to how weight-loss magnitude, energetic restriction degree, exercise dose and participant characteristics may all influence NEAT and/or NEPA (Silva et al., 2018). One explanation for a reduction in NEAT is that fatigue from the exercise intervention caused reciprocal increases in total time spent inactive when the individual would have usually been active (prior to starting the exercise intervention). That is, individuals could consciously or unconsciously perceive rest periods as reward for exercising, and factors, such as muscle soreness and general tiredness, could lower subsequent physical activity (discussed below).

### Inter-individual variability in exercise compensatory responses

It is well established that energy compensation for exercise interventions is variable between individuals (King et al., 2008). Compensatory responses, or the lack of response, account for the diversity in weight loss following fixed exercise loads. This could explain why some people are resistant to or susceptible to the theoretical weight loss associated with exercise. A problem with most studies evaluating the effectiveness of exercise on weight loss is that they tend to report the mean data only, which inadvertently masks the inter-individual variability. Expressing only the mean data fails to explore the variability in exercise-induced weight loss, which inherently prevents the opportunity to identify candidates for, and the characterization of, compensatory responses. Recent studies have highlighted the phenotypic responses as ‘poor’ and ‘good’ responders. The concept of exploring individual variability in response to fixed feeding or exercise interventions is not original. Claude Bouchard’s classic studies identified the variability in weight change in response to over-feeding interventions in twins (Tremblay et al., 1987; Bouchard et al., 1990). Bouchard was also the first to demonstrate large inter-individual variability (i.e., good and poor responders) in changes in maximum oxygen consumption (VO_2_max) in response to a fixed-load of exercise training (Bouchard et al., 1990). Levine has also proposed that individual variability in postural allocation (e.g., time spent sitting, standing) influences the predisposition to gain weight (Bouchard et al., 1990). Therefore, compensatory responses, and their associated variability, make some individuals susceptible to weight loss through exercise and render others resistant (Bouchard, 1995; Snyder et al., 1997; Weinsier et al., 2002; Blundell et al., 2003; Stubbs et al., 2004). Little is known regarding the variability in the volitional behavioral compensatory responses centered on decreasing non-exercise PA when engaging in an exercise intervention. This is despite several studies demonstrating decreases in NEAT when engaging in an exercise intervention (Blaak et al., 1992; Levine et al., 1999; Meijer et al., 1999; Westerterp, 2003). Further, this response has not always been observed, necessitating additional investigation behind potential mechanisms that may promote this compensatory response (Flack et al., 2018, 2020a; Martin et al., 2019).

### Behavioral compensatory responses

The two most relevant behaviors that contribute to the overall compensatory response to an exercise-induced energy deficit are changes in eating behaviors (influencing EI) and changes in non-exercise activity behaviors (influencing NEAT). Both behaviors are greatly influenced by motivation. Eating is targeted as an important behavioral compensatory response due to its potent contribution to energy balance, whereas physical activity tends to be overlooked. Therefore, while the Laws of Thermodynamics and Homeostasis propose feasible theoretical concepts of energy balance, they take little account of the motivations and psychological factors underpinning compensatory behavior, especially in the realm of NEAT. Indeed, these classic theories were originally based on physiological and metabolic processes associated with systems and states of steady internal, biological conditions (e.g., blood glucose; Newton’s 3rd Law - “To every action, there is always an opposite and equal reaction”). By nature of their automated, biological regulation, they tend not to take into account motivational control or individual variability.

A key issue is whether the human regulatory system is capable of selectively determining which, when, and the magnitude, of behavioral responses compensating for the exercise-induced energy deficit. The temporal profile of compensatory responses will inevitably influence weight loss through feedback mechanisms that may render the use of exercise a less efficacious method for weight management. For instance, immediate compensatory responses (i.e., present during the first few weeks), may resist initial weight loss (Flack et al., 2022), while more ‘delayed’ compensatory responses (i.e., exhibited after several weeks/months), may result in a plateauing of weight loss. This could lead to disappointment, reduced expectation or belief that exercise can result in the desired weight loss, lessened perceived behavior control and, perhaps, exercise drop out (Conroy et al., 2007). These reflective factors are considered “Type 2” processes in dual-process models of behavior (Brand and Ekkekakis, 2018). More pertinent to our hypothesis, however, are automatic “Type 1” processes (e.g., hedonic / appetitive pathways) at play that may work less visibly but with equal or greater force to undermine exercise behavior and lifestyle physical activity. For Type 1 behavioral compensatory responses to be enacted, there needs to be a priming and detection of internal signals (e.g., leptin), associated with any reductions in body weight and tissue composition (Wang et al., 2019). How an individual responds behaviorally to these signals may be in the form of a downstream motivational state (e.g., urges, cravings, impulses), as described in various models, such as the Affective-Reflective Theory of Physical Inactivity (ART) (Brand and Ekkekakis, 2018) or the COM-B System / Behavior Change Wheel (Michie et al., 2011). Alternatively, they can resist and overcome the signal-induced compensatory behavior, via Type 1 (e.g., counter-impulses) and Type 2 motivational control processes (e.g., action planning).

### Motivational nudges and brakes

Dual process models of exercise behavior, however, have resurrected the idea from Kurt Lewin about propelling and restraining forces (Brand and Ekkekakis, 2018) – the psychological analog to physical forces explained above. These fall roughly into two categories, internal (or endogenous) factors and external (or exogenous) factors. It is important to acknowledge that behaviors are strongly influenced by external factors or stimuli, sometimes referred to as nudges or cues. Similarly, the endogenously-generated urge or impulse to act on these factors will be moderated by motivational factors or counter-impulses such as restraint, inhibition–processes of self-control (Michie et al., 2011; Carver and Scheier, 2017). It is also worth bearing in mind that these nudges and signals, and associated urges and impulses, related to behavioral initiation, interplay with factors associated with suppressing or halting behaviors. The motivational endpoint might be the result of the preponderance of factors working to promote a behavior balanced against those working against it (Brand and Ekkekakis, 2018). Unfortunately, those in Western societies, but increasingly in non-Western as well, are surrounded by environments that encourage a sedentary lifestyle (e.g., many prompts to stop and watch a video clip), with few cues or nudges to be active.

Indeed, there is an asymmetry of motivational signals to start and stop eating or moving. From behavioral “nudges” (i.e., “go”/want) and “brakes” (i.e., “stop”/do not want) point of view, it is commonly thought that humans are predisposed to promote a positive energy balance and to protect against a negative energy balance (Chakravarthy and Booth, 2004). Table 2 shows the imbalance between the internal cues associated with eating, PA, sedentary activities (e.g., television), and sleep. In relative terms, we have strong impulses or urges to initiate the motivation to eat (e.g., hunger), and weaker internal cues (e.g., fullness) to stop (brake) eating (Hofmann et al., 2012; Stevenson et al., 2015). With PA and exercise, there are relatively weak internal cues or urges to be active (Stults-Kolehmainen et al., 2020), especially in an environment that encourages and enables inactivity. However, there are strong internal cues associated with the urge to stop exercising, for example, from muscle pain or the different facets of fatigue (Hartman et al., 2019). There are large inter-individual differences (Stevenson et al., 2015; Stults-Kolehmainen et al., 2021). Nevertheless, there is an imbalance of impulses and counter-impulses when considering the factors associated with behavioral compensation in response to exercise. Reinforcement for food is so strong because the combination of pleasure from eating is great (positive reinforcement) and strong displeasure (hunger) is concomitantly reduced. With exercise, this pattern of reinforcement is not as universal as food reinforcement, but can be prevalent, especially among people dependent on exercise (Stults-Kolehmainen et al., 2020). In this condition, deprivation of activity causes significant tension, which is only relieved with exercise (Marques et al., 2019). When considering the compensatory responses to exercise, this imbalance of relative potency and contributions of internal brake-and urges-signals could also have an impact on the desired method for weight loss.

**Table 2:** The relative imbalance of impulses and counter-impulses (i.e., internal nudges and brakes/constraints) to either promote or stop eating, physical activity, sedentary, and sleep behaviors (typically observed across the adult population), as varying in strength by hedonic motivation.

	Impulses	Counter-impulses	Pleasure provided (positive reinforcement)	Displeasure removed (negative reinforcement)
**Eating**	Strong	Weak (fullness)	Strong	Moderate (hunger)
**Physical activity (incidental, NEAT)**	Weak	Strong	Weak*	Weak*
**Exercise**	Weak	Strong	Weak*	Weak*
**Sedentary activities (e.g., television)**	Strong	Weak	Moderate	Weak to moderate
**Sleep**	Strong	Weak	Moderate	Strong (fatigue)

*PA and exercise are typically thought to provide relatively weak amounts of pleasure and remove relatively small amounts of displeasure, but this is not universal as some individuals experience a high amount of pleasure from exercise.

The strongest impulses and urges should come from activities that result in the provision of higher pleasure combined with removal of displeasure. Sleep and eating, therefore, have the greatest urges, theoretically. Furthermore, some experience high amounts of tension from a lack of exercise that results in negative reinforcement when exercise is engaged [see (Stults-Kolehmainen et al., 2022b) for a broader discussion].

### Role of rewards and motivation in energy compensation

As noted, the compensatory response to an exercise-induced energy deficit is highly variable, potentially due to the ‘sensitivity to reward’ reflecting a trait where some individuals are more tuned for reward from activities, such as exercise and eating, than others (Davis and Woodside, 2002). Evidence indicates that vigorous exercise and eating behaviors, particularly food restriction, stimulate brain circuitry which are associated with reward and dependence (Bergh and Sodersten, 1996). Indeed, exercise has been shown to increase perceived ratings of pleasantness for a range of highly-palatable foods (Lluch et al., 1998, 2000). Our team has also demonstrated acute exercise increases one’s attentional processing toward food (an important component in the development of behavioral reinforcement) and that declines in fat-free mass from an aerobic exercise program predict increases in food reinforcement (Flack et al., 2020b, 2022). These reward-driven food choices, as well as misperceptions about the rate of intake (number of calories consumed) relative to the energy cost of physical activity (energy expended), could also drive compensatory responses, which in turn, result in lower than expected weight loss (Blundell et al., 2003). Processes of motivation have generally been overlooked as compensatory mechanisms explaining changes in non-exercise physical activity, but recent data and theories have implicated motivation in physical activity compensation and sedentary behavior. In King’s review, motivation was tangentially discussed as a mechanism influencing NEAT (King et al., 2007). It was posited that the motivational *drive to be inactive* is the key mechanism in place to promote decreases in physical activity outside the exercise intervention. The role of motivation as a compensatory mechanism was also explored in a recent systematic review (Swelam et al., 2022). These authors specifically listed 4 factors as impairing physical activity in the face of exercise training: (1) lack of motivation, (2) drive to be inactive, (3) fear of overexertion, and (4) autonomous motivation. The major theories of motivation (e.g., Self Determination Theory) do not seem able to accommodate the diversity of aforementioned motivational constructs, thus necessitating novel and innovative research questions and theories to better explain the implications of shifting motivation in energy expenditure compensation specific to decreases in NEAT (Ryan and Deci, 2000).

Exercise is highly rewarding for some individuals, and is generally agreeable for most individuals, giving rise to the notion of exercise as a reinforcing behavior controlled by central dopamine signaling and termed “exercise reinforcement” (Cheval et al., 2018; Flack K. et al., 2019). Indeed, exercise has reinforcing properties in that exercise dependency has been demonstrated in both humans (Chan and Grossman, 1988; Chapman and De Castro, 1990; Belke, 1997; Holden, 2001) and rodents (Iversen, 1993; Belke, 1997, 2000; Lett et al., 2000). Recent cross sectional data have demonstrated that adults who find aerobic exercise highly reinforcing are more likely to meet PA guidelines for aerobic exercise while those who find resistance-type exercise more reinforcing are more likely to meet PA guidelines for both muscle-strengthening and aerobic exercise (Flack et al., 2017). Thus, it appears the reinforcing value of exercise is an important determinant in the choice to engage in exercise frequently enough to meet the Physical Activity Guidelines for Americans (Epstein et al., 1999; Barkley et al., 2009).

This has given rise to a growing body of literature demonstrating that exercise reinforcement can be increased through the process of “incentive sensitization,” a theory originally proposed to explain drug addiction (Robinson and Berridge, 1993). Incentive Sensitization theory states that behavioral reinforcement is increased through repeated exposures by producing neuroadaptations that increase the craving of the behavior (Salvy et al., 2009). After repeated exposures to a stimulus, a ‘sensitization’ or hypersensitivity to the motivational effects of the stimulus follows. The result is an increased reinforcing value of the stimulus relative to a competing alternative. In this light, an exercise intervention that exposes individuals, repeatedly, to bouts of exercise can produce increases in exercise reinforcement (Flack et al., 2019a,b, 2021; Ufholz et al., 2019). However, recent evidence by our group indicates changes in exercise reinforcement from pre- to post-exercise intervention are not correlated with the overall compensatory response to that exercise intervention (Flack et al., 2018, 2019a, 2020a, 2021). This could be due to the large individual variability in the compensatory response noted above, where some individuals may increase their exercise reinforcement and have greater motivation to exercise while others do not. Conversely, differences in exercise reinforcement and the desire to be physically active outside the exercise intervention (free-living PA) may be two very different concepts. Furthermore, it is certainly possible to be highly motivated to exercise but also very motivated to rest (i.e., reduced physical activity) after the exercise bout. It is also possible that an athlete will desire to rest more in order to train harder at a later time. These may be likely scenarios if the motivation to exercise is high enough to promote extremely high levels of exercise.

### WANT model

A recent conceptual framework, the WANT model (Wants and Aversions for Neuromuscular Tasks), may be better positioned to describe motivational factors as compensatory mechanisms for exercise training (Stults-Kolehmainen et al., 2020). The basic tenet of the WANT model is that humans have “wants” for movement that vary from moment to moment. These desires are loosely coupled and work asymmetrically with desires to rest or be sedentary. For instance, it is possible for the “want for movement” to remain stable and the “want for rest” to increase (or *vice-versa*). In general, the ratio of these two constructs is continually changing but infrequently equal. These “wants” vary in intensity from weak desires to strong urges and cravings for movement, when they are typically felt subjectively and often acted upon (Stults-Kolehmainen et al., 2022a). Collectively, wants, desires, urges and cravings are considered “affectively-charged motivation states” (ACMS), impulses for activity that are often felt as tension in conditions of deprivation or excess (Kavanagh et al., 2005; Stults-Kolehmainen, 2022).

In this way, they vary by previous behaviors - i.e., when sitting for a long time (deprived of activity) urges for movement rise (Stults-Kolehmainen et al., 2021). Likewise, with excessive movement, like maximal exercise, desires to move fall, and urges to stop and rest rise (Stults-Kolehmainen et al., 2021; Taylor et al., 2022). These desires, consequently, seem to flow in processes of satiation and accommodation, similar to desires for other rewarding activities, like eating, drinking, smoking, using drugs, etc. (Redden, 2015). This has prompted some researchers to refer to the desires for movement as “appetence” (Ferreira et al., 2006; Redden, 2015; Robinson et al., 2016; Stults-Kolehmainen, 2022). Similar to these other rewarding activities, appetence for movement increases with restriction and results in mental and physical tension, such as jitteriness and fidgeting, until the desire is consummated, which results in a sharp decrease in appetence (i.e., feel “fulfilled”, “satisfied”, or “exertiated’’, and do not want any more) (Stults-Kolehmainen et al., 2022a). Such an appetite is likely propelled by both positive reinforcement (i.e., pleasurable aspects of exercise) and negative reinforcement (i.e., relieving tension from disuse of muscle and / or from the drive to be active) (Stults-Kolehmainen, 2022).

Work in this area has been stymied by a lack of useful measurement tools, but new developments in the measurement of motivation states (CRAVE scale: Cravings for Rest and Volitional Energy Expenditure) have facilitated collection of new data (Stults-Kolehmainen et al., 2021). This scale was developed with 9 studies, demonstrating superior psychometric properties, such as high face, construct and convergent validity, good reliability and intra-class correlations, indicating that the scale measures states and not traits (Stults-Kolehmainen et al., 2021, 2022a; Budnick et al., 2022; Filgueiras et al., 2022). With use of the CRAVE, it was demonstrated that appetence for movement and rest rises and falls continuously in response to a variety of stimuli and situations (Stults-Kolehmainen et al., 2021, 2022a). With a maximal treadmill test, there were large effect sizes observed for increases in the desire to rest and decreases in the desire to move. With both a passive and active sedentary activity, desires to move increased after 45 min and desires to be sedentary decreased (Stults-Kolehmainen et al., 2021, 2022a). CRAVE is also associated with body position, such as laying down, sitting, leaning on something (e.g., a wall), standing, and walking (Budnick et al., 2022). Desires to move and rest also predict intentions to move in the next 30 min. Furthermore, a majority of people have a biorhythm for movement motivation (Budnick et al., 2022). These data stand in contrast to theories that promote the idea that humans are primarily driven to rest and save energy (Cheval and Boisgontier, 2021).

### Fatigue and pain: Implications in energy compensation

As suggested above, fatigue and soreness could account for both short-and long-term compensatory responses that reduce EE after acute and chronic bouts of training. Fatigue serves as a feedback response and causes increased inactivity when the individual would have been otherwise active to resist returning to an energy balance (Stubbs et al., 2004). A natural reaction to fatigue is to rest and to choose activities that require minimal energy expenditure (Cheval and Boisgontier, 2021). For example, retiring to bed earlier in the evening could influence the impact of the acute energy deficit created by exercise. In this way, exercise-induced fatigue is an underlying mechanism promoting decreases in non-exercise physical activity (Wlodek and Gonzales, 2003; Cheval and Boisgontier, 2021). Importantly, exercise-induced fatigue is a complex phenomenon with various peripheral and central processes, including metabolite accumulation, depletion of fuel reserves, neuromuscular damage and changes in neurotransmitter levels (Lambert, 2005).

Noakes et al. (2004) were among the first exercise physiologists to propose that fatigue is an emotional state, rather than a physical state, and that the sensation of fatigue is an interpretation of the current level of activity as well as future exercise capacity. If this is the case, we know that emotional and motivational states can be influenced by attention, perception, interpretation, and memory (Cheval and Boisgontier, 2021). Specific exercise experiences are associated with memories of energy and fatigue, causing one to continually estimate their reserves and tolerance levels. In the case that fatigue is assessed as high and tolerance is low, it would result in a greater sense of effort and impaired motivation for movement (Iodice et al., 2017). For instance, Iodice et al. (2017) demonstrated that under conditions of fatigue, people prefer lower-effort behaviors. Prolonged and/or intense fatigue may even lead to sensations of hedonic dread for physical activity (Williams and Bohlen, 2019; Stevens et al., 2020), otherwise known as movement aversion or ‘diswant’ (Stults-Kolehmainen et al., 2020, 2022b). Such may be the case with athletes experiencing burnout and associated loss of motivation (Kentta and Hassmen, 1998; Poscente and Irvine, 2002; Oldervoll et al., 2003; King et al., 2007; Blaney et al., 2010; Purvis et al., 2010; Van Oosterwijck et al., 2010; Yoon et al., 2013; Legrand et al., 2018). These mental processes can thus be considered important mediators on the compensatory response, directing decision-making to reduce or enhance physical activity levels (St Clair Gibson et al., 2003).

Pain is also an important psychosomatic sensation contributing to compensatory responses. Delayed-onset muscle soreness (DOMS) due to exercise, for example, can produce pain and discomfort associated with muscle tenderness, stiffness, and weakness (Cleary et al., 2005). DOMS interferes with physical activity levels since any movement or palpation may exaggerate the smallest increase in pressure and stimulate the pain receptors, thus reducing muscle function (Smith, 1991). Individuals classified as obese report physical activity and exercise as a painful experience, causing severe soreness and stiffness, and often perceive their bodies as too heavy and incapable of more exercise (Danielsen et al., 2016). Individuals with obesity associate physical activity with pain and anxiety (Mæhlum et al., 2012) that can interfere with physical activity levels (Ginis et al., 2003; Dobkin et al., 2006; Law et al., 2010, 2013). In the worst case scenario, repeated or high-intensity exposure to pain during exercise may even result in kinesiophobia (Glaviano et al., 2019). It is important to note that in highly motivated populations, such as athletes or perhaps those with anorexia nervosa or muscle dysmorphia, sensations of pain and fatigue may be routinely bypassed or disregarded in the effort to maintain very high levels of activity and performance (Marcora and Staiano, 2010). Finally, it should be noted some exercise routines, like Curves, were designed to minimize excessive soreness by limiting eccentric contractions (Kerksick et al., 2009). The concept is that less damaging exercise is better tolerated, more acceptable, and more sustainable. Likewise, short sprint interval training (sSIT) (de Sousa et al., 2018; Boullosa et al., 2022; Filgueiras et al., 2022; Metcalfe et al., 2022) involves very short bouts of exercise (5–6 s) that do not produce painful sensations. In the case of Curves, the degree of compensation is unknown. SSIT, on the other hand, is too short to result in important reductions in body fat, but interestingly, it may result in enhanced desire to move and be active (Filgueiras et al., 2022).

### Influence of sleep and sedentary behaviors on energy compensation from exercise

Any discussion about the effects of physical activity and exercise on weight management would be remiss if it did not consider sedentary behaviors and sleep, which account for most of the 24-h activity cycle (Rosenberger et al., 2019). Rest behaviors are not the inverse of physically active ones. In fact, as discussed previously, one may be high in both sedentary and active behaviors on the same day (Thompson and Batterham, 2013). Physical activity, sedentary behavior, and sleep are under control from distinct, but interacting, neurobiological systems (Dishman et al., 2006; Harrington, 2012). According to the WANT model, motivation for movement and sedentary behaviors is asynchronous (Stults-Kolehmainen et al., 2020). One might be motivated to move and sleep or be sedentary at the same time, with the same intensity. There may also be a total absence of desire to move or rest. Their correlation is moderate (*r*’s typically −0.6 to −0.7; Stults-Kolehmainen et al., 2021, 2022a). The key issue is whether exercise training enhances desires to rest, be sedentary or, more specifically, to sleep (e.g., tiredness, sleepiness). This is a different question than if exercise training reduces the desire to move or be active.

The desire to sleep or nap is one of the most common desires humans possess (Hofmann et al., 2012). Sleep, however, is an understudied variable in exercise and weight loss research. Both epidemiological and experimental evidence demonstrate that sleep quantity and quality may influence body weight and energy balance regulation (Garaulet et al., 2011; Markwald et al., 2013; Wirth et al., 2015; Sun et al., 2016; Park et al., 2018; Liu et al., 2019; Sa et al., 2020). Experimental studies have found that reducing sleep duration from 9 h/night to 5 h/night alters metabolic energy expenditure while promoting insulin resistance and weight gain (Jung et al., 2011; Markwald et al., 2013; Eckel et al., 2015). Although most of these studies have focused on how sleep timing and duration may influence EI, there is also evidence that sleep and NEAT may be linked (Lambiase et al., 2013). Thus, understanding how aspects of sleep are influenced during exercise trials is imperative, particularly in exercise studies focused on the impact of the intervention on bodyweight.

Exercise and sleep have a dynamic relationship (Youngstedt and Kline, 2006; Kline, 2014; Ash et al., 2021). It is likely that sleep and exercise are synergistic with better sleep helping to support exercise behavior and engagement in exercise helping to promote better sleep. For instance, Lambiase et al. (2013) found that greater sleep efficiency was associated with greater total daily physical activity and greater moderate to vigorous physical activity (MVPA) the following day. Those who sleep well are also likely more motivated to move and exercise (Hong and Dimsdale, 2003; Baron et al., 2013), perhaps by lessening fatigue and enhancing feelings of energy, arousal, and motivation states to move (Nicholson et al., 1984; Frederick et al., 2021). Thus, the common suggestion of reducing sleep duration when looking to increase total daily EE may be counterproductive because of the negative effects on metabolism, psychological well-being, and motivation (Chennaoui et al., 2015).

To date there is limited evidence on what happens to aspects of sleep when initiating exercise. When an individual who is previously sedentary begins an exercise intervention, the time spent engaging in the exercise must replace time spent engaging in a previous behavior. Because there is only a finite amount of time in day, it is possible that time spent engaging in exercise replaces sleep time apart from other sedentary activities. Interestingly, in the study by Lambiase et al. (2013) lower sleep time was associated with more minutes of MVPA across the week. Creasy et al. (2022a) also found that adherence to MVPA recommendations for weight management (300 min/wk) was associated with lower time in bed and lower sleep duration. Further, in a study that examined the effect of morning vs. evening exercise on bodyweight, morning exercisers woke up earlier in the morning to engage in their exercise, thereby reducing sleep duration (Creasy et al., 2022b). Evening exercisers went to bed earlier and woke up later, thereby, increasing sleep duration. Thus, the time of day of exercise may be a crucial factor in bodyweight management interventions. It is critical that future studies examining the effect of exercise on bodyweight also measure sleep to better understand compensatory mechanisms.

### Future studies

Very few studies directly connect the concept of motivation states for movement (e.g., appetence) with energy expenditure and/or compensatory effects (Stults-Kolehmainen et al., 2021). To investigate these potential associations, the following key areas should be considered:

1. Track the subjective experience of wanting/desiring to move (and be sedentary or sleep) with acute bouts of exercise training at various intensities (e.g., moderate, vigorous), and durations, starting with the public health dosage (150 min/week of MVPA) and then progressively increasing this to 300 min/week, the recommendation associated with greater impact on weight loss/maintenance (Flack et al., 2018, 2020a).
2. Conduct longer training studies, up to a minimum of 6 months, that evaluate the relationship between motivation states and energy compensation.
3. Attempt to modulate motivation states with training studies that aim to mitigate fatiguing and painful sensations during or after exercise, perhaps by: (a) lowering intensity at the end of the training session, (b) through proper exercise ramp-up/progression, or (c) minimizing concentric contractions or glycolytic activity. Alternatively, attempt to maximize enjoyment and pleasure, as liking is usually matched with wanting (Stults-Kolehmainen et al., 2020).
4. Implement controlled exercise training interventions that monitor, at the same time, non-exercise physical activity, sedentary time and sleep.
5. Explore mechanisms that may mediate processes of motivation and physical activity in response to exercise training, such as orexin /hypocretin, which has been implicated in the regulation of appetite, sleep, and arousal (Sakurai, 2005).

### Conclusion

The main premise of the current paper is that humans have an appetite for movement (or rather, “appetence”) that may be altered with an exercise training program and the related changes in lifestyle and daily activities. This has long been hypothesized for eating behaviors, though the effects of exercise on appetite for food and hunger are equivocal. At the current time, it is not known how exercise training impacts the desire or want to move and be active. There is some evidence, though conflicting, that exercise training impacts lifestyle physical activity outside of the training program, typically by lowering it, but in some cases enhancing it (Foster-Schubert et al., 2012). It may be the case that these effects have been poorly explored due to difficulties in the detection of these behavioral processes in play and the large inter-individual variability in their response to exercise. New developments in motivational theory (Stults-Kolehmainen et al., 2020, 2021) and instrumentation (Stults-Kolehmainen et al., 2021) may spur greater efforts to investigate these phenomena.

## Data availability statement

The original contributions presented in the study are included in the article/supplementary materials, further inquiries can be directed to the corresponding author.

## Author contributions

MS-K, NK, and KF developed and conceived the manuscript. MS-K, NK, KF, SC, SK, DB, and VC completed the writing. All authors contributed to the article and approved the submitted version.

## Funding

Portions of this work was funded by the United States Department of Agriculture-Agricultural Research Service (USDA-ARS) Project 3062–51000-051-00D and National Institutes of Health P30GM127211 of the National Institute of General Medical Sciences. DB was supported by Grant RYC2021-031098-I funded by MCIN/AEI/10.13039/501100011033, by “European Union NextGenerationEU/PRTR,” and by a productivity research grant (PQ1D) from CNPq (Brazil).

## Conflict of interest

The authors declare that the research was conducted in the absence of any commercial or financial relationships that could be construed as a potential conflict of interest.

## Publisher’s note

All claims expressed in this article are solely those of the authors and do not necessarily represent those of their affiliated organizations, or those of the publisher, the editors and the reviewers. Any product that may be evaluated in this article, or claim that may be made by its manufacturer, is not guaranteed or endorsed by the publisher.

## Author disclaimer

The funding sources of this study had no involvement in data collection, analysis, interpretation or the decision to submit for publication. Mention of trade names, commercial products, or organizations does not imply endorsement from the U.S. government. USDA is an equal opportunity provider and employer. The results of the study are presented clearly, honestly, and without fabrication, falsification, or inappropriate data manipulation.
